# Workplace musculoskeletal problems in occupational therapy students

**DOI:** 10.1186/s12889-021-10653-8

**Published:** 2021-04-06

**Authors:** Joanne Morabito, Stefania Penkala, Kristy Coxon

**Affiliations:** 1 School of Health Sciences, Western Sydney University, Sydney, Australia; 2 Translational Health Research Institute, Western Sydney University, Sydney, Australia

**Keywords:** Occupational therapy, Workplace health, Students, Work-based training, Musculoskeletal problems

## Abstract

**Background:**

Workplace musculoskeletal disorders are the leading cause of morbidity and disability in the Australian workforce. Over one in five occupational therapists report workplace musculoskeletal disorders, with almost half reporting workplace musculoskeletal symptoms. In other health professions, students and novice clinicians (≤5 years practice) experience greater risk but little is known about occupational therapy students.

**Methods:**

In this cross-sectional study, a survey including the self-reported Standardised Nordic Musculoskeletal Questionnaire was administered to occupational therapy students post work-based training. Musculoskeletal problems were defined as aches, pains, numbness or discomfort. Questions explored body sites affected, prevalence, impact on activity, need for medical assistance, demographic and workplace information. Prevalence was reported using descriptive statistics. Factors associated with workplace musculoskeletal problems over the previous 12 months and last 7 days were examined using logistic regression modelling.

**Results:**

Response rate was 53% (*n* = 211/397). One-third of respondents (33.6%, *n* = 71/211) reported a workplace musculoskeletal problem over 12 months. Nearly half (47.9%, *n* = 34/71) of these students reported a problem over the last 7 days. Neck was the most commonly affected area reported for musculoskeletal problems over the past 12 months (24.2%, *n* = 51/211) and shoulder areas affected over the past 7 days (10.9%, *n* = 23/211). Musculoskeletal problems preventing daily activities were reported most commonly from lower back problems over 12 months (23.9%, *n* = 17/71) and for shoulder problems over the last 7 days (21.9%, *n* = 7/32). Shoulders and knees were the most common body areas requiring medical attention. Previous musculoskeletal problems and female gender were associated with reported problems over 12 months and last 7 days (*p* < 0.05). Non-standard joint mobility (OR = 3.82, *p* = 0.002) and working in psychosocially focused caseloads (including mental health or case management) (OR = 3.04, *p* = 0.044) were also associated with reporting musculoskeletal problems over the last 7 days.

**Conclusions:**

One in three occupational therapy students already experience workplace musculoskeletal problems impacting daily activities and requiring medical assistance prior to graduation. High prevalence of musculoskeletal problems in this study calls for educators and researchers to find sustainable strategies to address these problems, with particular consideration to the impact of previous disorders and working in psychosocially focused caseloads on musculoskeletal health.

## Background

Globally, workplace musculoskeletal disorders are the primary cause of morbidity and disability in any workforce [1], and impact on the individual’s quality of life, everyday function and working ability [2]. In Australia, 3,60,180 serious workplace musculoskeletal disorder claims were lodged between years 2009 to 2014 [1]. Healthcare and social assistance occupations reported the highest prevalence (18%) and frequency rate (7.1 claims per million hours worked) of workplace musculoskeletal disorder claims during this period [1]. Subsequent loss of function, poor job satisfaction, reduced productivity and inability to participate in work activities can lead to workforce shortages in healthcare through absenteeism [3]. Overall musculoskeletal claims were estimated to cost 61.8 billion in year 2012–13 in Australia [4].

Research about workplace musculoskeletal disorders in health professionals has focused heavily on nurses and physiotherapists [5–7]. However, other health professionals including occupational therapists are also at risk [8]. Workplace musculoskeletal disorders experienced by occupational therapists have been investigated in several studies [9–16]. Prevalence of disorders varied from 23% [9] to 63% [15] in American and Australian studies with sample sizes between 46 and 192. Higher prevalence in the Australian study maybe in part explained by using a survey based on the Standardised Nordic Musculoskeletal Questionnaire (SNMQ), and identifying musculoskeletal problems based on symptoms (i.e. aches and pains) in contrast to the American study reporting more substantive but less prevalent disorders. Similarly, body areas affected by workplace musculoskeletal disorders in occupational therapists varied between studies including the neck (11.1–32.8%), lower back (27.2–50%), shoulders (23.3–26.9%), hand/wrist/fingers (10.5–24.5%) and upper back (18.4–22%) [13, 14].

Risk factors have primarily focused on ergonomic or biomechanical factors including load handling, forceful exertions, poorly designed work environments, static and awkward postures and high task repetition [17]. However, personal risk factors including female gender, poor work practices, co-morbidities, reduced rest, poor nutrition and decreased fitness level may also contribute [18]. Inherent differences in body size and physical capacity place females at greater risk of workplace musculoskeletal problems in physically demanding health professions [19]. The high prevalence of disorders in females may further be explained by the predominantly female healthcare workforce [20].

Emerging evidence links psychosocial risk factors to the prevalence of workplace musculoskeletal problems [10]. Psychosocial risk factors may include lack of job control/autonomy, high job demands, lack of social/emotional support and role conflict or ambiguity [10]. In healthcare professions, time constraints and productivity pressures of caring for people may heighten the risk of workplace musculoskeletal disorders [21]. Further to this, health professionals are routinely exposed to emotionally and psychologically challenging and confronting situations such as recounts of trauma, tragedy, illness and disease [10]. Dealing with stressful situations heightens emotional and psychological workplace demands, increasing vulnerability to burnout [22] and resultant sick leave [23]. While supportive line managers, and/or co-workers, and self-efficacy help address disproportionate workplace stress [24], without adequate social supports, self-care and strategies for emotional well-being, susceptibility to injury may increase. Although links between psychosocial risk factors and musculoskeletal health are emerging, the full impact is not yet known.

Evidence indicates 23 to 63% of occupational therapists experience workplace musculoskeletal disorders or problems [9–16]. Prevalence of similar problems in occupational therapy students is scarce. A study conducted in the last decade investigated upper body musculoskeletal problems in Australian occupational therapy students [25]. Three quarters of students reported a musculoskeletal problem in at least one upper body region in 12 months [25]. However lower body problems, and problems related to work-based training remain unknown. Evidence suggests students and early career practitioners (≤5 years practice) are at greater risk of workplace musculoskeletal disorders [6, 26, 27]. This is consistent with student studies in nursing (80%) [28], medial science (35%) [2] and dentistry (61%) [29], as well as, disorders reported in early career occupational therapists (63%) [15] and podiatrists (45%) [26]. The aim of this study was to 1) describe prevalence of workplace musculoskeletal problems in occupational therapy students over a 12 month and 7 day recall period, and 2) explore factors influencing reporting of these workplace musculoskeletal problems.

## Methods

### Study design

A cross-sectional study design was employed to capture data via survey from occupational therapy students post placement. Ethics approval was obtained from the Western Sydney University Human Research Ethics Committee (protocol: H9563). Consent was implied if students completed the survey.

### Participants

Participants were students in the Bachelor of Occupational Therapy program (4-year program) at a university in New South Wales, who were enrolled in a practice unit in 2017. Invitations were sent via student email or given to students in practicum classes. Students not enrolled in a practice unit, or who failed a threshold assessment prior to work-based training, were excluded.

### Data collection

Surveys were completed anonymously by occupational therapy students to investigate the prevalence of workplace musculoskeletal problems. All data were self-reported and collected via an online survey using Qualtrics (or paper-based copy) post completion of their work-based training. Surveys were completed online, unless there was an internet problem where students were offered the same survey to complete on paper. Paper surveys were only completed by a small number of students (< 20%). Data were collected from November 2017 to March 2018.

#### Personal and placement information

Data were collected on age, weight, height, gender, year of study, placement hours, previous musculoskeletal problem/s, joint mobility, and quality of working posture. Joint mobility was self-reported as hypermobile, hypomobile, or within normal limits, and working posture on placement reported as poor, good or excellent. Body mass index was calculated from height and weight. Students were asked to report the primary patient case type seen on their placement. Occupational therapists typically refer to this as their patient caseload. Caseloads were dichotomised into two broad categories based on the primary caseload focus; (1) physical or (2) psychosocial. Physically focused caseloads included practice areas such as physical rehabilitation, aged care and medical/surgical. Psychosocially focussed caseloads included practice areas such as mental health, occupational rehabilitation and case management. Perceived joint mobility was dichotomised into standard (within normal limits) and non-standard (hyper- or hypo-mobility). Year of study (early program Years 1 and 2 or late program Years 3–4) and working posture (Poor/Good or Excellent) were also dichotomised for analysis.

#### Standardised Nordic musculoskeletal questionnaire (SNMQ)

Musculoskeletal data were collected using the self-reported SNMQ. The SNMQ has been previously used to collect workplace musculoskeletal disorder data in nurses [30], dentists [31], podiatrists [32], laboratory technicians [33] physiotherapists [34] and medical science students [2]. The SNMQ has good test-retest reliability (77 to 100% agreement) and validity (80 to 100% agreement) when compared to clinical history [35].

The SNMQ included questions about musculoskeletal problems experienced during work-based training, their impact on participation in daily activities and/or need to seek assistance from a physician or health professional. Musculoskeletal problems were defined as an ache, pain, numbness or discomfort in different body areas over a 12 month and 7 day recall period [36]. Participants were able to report concurrent problems at different body sites within each recall period. Work-based training included all practice education including university-based skill practicums and occupational therapy work-based training (also referred to as placement) external to the university.

### Sample size

All 397 occupational therapy students enrolled in practice units were invited to complete the survey. Power was calculated post-hoc. With an expectation of one third of respondents reporting a workplace musculoskeletal problem, a total sample size of 202 would estimate the proportion of problems in the population to the accuracy of ±6.5%.

### Data analysis

Data from paper surveys were manually entered into Qualtrics by the research team. Survey data were exported from Qualtrics into a Microsoft excel spreadsheet (Version 97–2004) and imported directly into IBM Statistical Package for Social Sciences (Version 25 for Macs software) for statistical analysis. Demographic characteristics, common body areas and nature of workplace musculoskeletal problems were characterised using descriptive statistics. Mean and standard deviation were reported when variables were normally distributed, and median and interquartile range (IQR) reported when not. Categorical data were described using frequency and percentage to define sample proportions. Students needed to answer survey questions to at least the screener question “*In the last 12 months, have you experienced any workplace musculoskeletal problem(s) associated with your workplace activities (an ache, pain or trouble) as an Occupational Therapy Student?*” to be included in the statistical analysis. It was not possible to determine if students had experienced musculoskeletal problems or not if this question was unanswered, and therefore, the attempt was deemed invalid.

Regression modelling was used to explore factors associated with the presence of workplace musculoskeletal problems over 12 months and 7 days. A correlation matrix was used to examine relationships and possible collinearity between variables [37] prior to regression modelling. Each variable was analysed initially in an unadjusted model. All variables with an association of *p* < 0.25 in their unadjusted model were included in an initial adjusted model. Backwards stepwise elimination was used until only significant variables remained to determine final models explaining prevalence of workplace musculoskeletal problems in occupational therapy students over 12 months and 7 days [38]. Significance was set at 0.05.

## Results

### Participants

A total of 236 out of 397 occupational therapy students enrolled in practice units in 2017 responded to the survey. Of these 25/236 were excluded, as the screener question about prevalence of workplace musculoskeletal problems was not answered or the student abandoned the survey prior to reaching this question. Taking this into consideration, the study had a 53% adjusted response rate (number (*n*) = 211/397). Missing data for problems reported at different body regions was less than 2% and were treated as no problems. One case from the 12 month data and two cases from the 7 day data were removed as the participant/s did not specify the body area of problems. Response rates were highest in students’ final year of study (97%, *n* = 77/79; Table 1), followed by first year of study (79%, *n* = 93/118). The mean age of students was 23.6 ± 6.1 years, and the majority identified as female (82.9%). Over one-third (38.9%) reported a previous musculoskeletal problem. Mean body mass index was 23.8 ± 4.1.

**Table 1:** Participant characteristics

Characteristics (*n* = 211)	
Age, mean (SD)	23.6 (6.1)
Body mass index, mean (SD)	23.8 (4.1)
Gender, n(%)	
Male	36 (17.1)
Female	175 (82.9)
Year of study, n(%)	
Year 1	93 (44.1)
Year 2	7 (3.3)
Year 3	34 (16.1)
Year 4	77 (36.5)
Hours of work-based training, median (IQR)	180 (76–400)
Percentage time in, median (IQR)	
Face to face physical activities with clients	20 (10–30)
Face to face communication with clients	30 (20–40)
Clinical observation	20 (10–30)
Administrative/desk activities	20 (10–30)
Other	0 (0–0)
Number of clients treated during placement, median (IQR)	20 (10–40)
Working posture, n (%)	
Poor	75 (35.5)
Good	127 (60.3)
Excellent	9 (4.3)
Main workplace position, n (%)	
Standing	13 (6.2)
Sitting	35 (16.6)
Predominantly standing and sitting	61 (28.9)
Predominantly standing and walking	60 (28.4)
Predominantly sitting and walking	38 (18.0)
Other	4 (1.9)
Chair type during seated activities with clients, n (%)	
Stationary non-adjustable chair	137 (64.9)
Mobile chair on wheels but non-adjustable	10 (4.7)
Mobile chair on wheels and adjustable	40 (19.0)
Not applicable	24 (11.4)
Chair type when participating in administrative duties, n (%)	
Stationary non-adjustable chair	36 (17.1)
Mobile chair on wheels but non-adjustable	18 (8.5)
Mobile chair on wheels and adjustable	154 (73.0)
Not applicable	3 (1.4)
Primary focus of caseload, n (%)	
Paediatrics	37 (17.5)
Aged care	54 (25.6)
Rehabilitation	44 (20.9)
Mental health	29 (9.0)
Other	57 (27.0)
Hand dominance, n (%)	
Right	179 (84.8)
Left	26 (12.3)
Ambidextrous	6 (2.8)
Perceived joint mobility, n (%)	
Within normal limits	150 (71.1)
Hypermobile	50 (23.7)
Hypomobile	11 (5.2)
Performs exercises to improve posture on work-based training, n (%)	
Yes	66(31.3)
No	145(68.7)
Works with clients outside of studies, n (%)	
Yes	74 (35.1)
No	137 (64.9)
Previous musculoskeletal problem in last 12 months, n (%)	
Yes	82 (38.9)
No	129 (61.1)

*SD* standard deviation, *IQR* interquartile range, *n* = number

### Work-based training

Participants completed a median of 180 (IQR = 76–400) hours of work-based training in 2017. During work-based training, students spent 30% of their time in face-to-face communication with clients. Stationary, non-adjustable chairs were the most common seating used during seated activities with clients (64.9%; *n* = 137/211). Adjustable, mobile chairs on wheels were the most common seating used in administrative duties (73.0%; *n* = 156/211). Most occupational therapy students reported having a good working posture (60.2%; *n* = 127/211), with 71.1% (*n* = 150/211) reporting their perceived joint mobility was within normal limits. Of the total sample, 31.3% (*n* = 66/211) reported performing exercises to improve posture while on work-based training. Of these 19.7% (*n* = 13/66) reported participating in yoga or pilates, 45.5% (*n* = 30/66) reported stretching, 28.8% (*n* = 19/66) reported going to the gym and 15.2% (*n* = 10/66) reported standing, walking and/or running.

### Prevalence and body areas of workplace musculoskeletal problems

One in three occupational therapy students (33.6%; *n* = 71/211) reported a workplace musculoskeletal problem in the last 12 months. In the more immediate timeframe of the last 7 days, 16.1% (*n* = 34/211) of the whole sample reported a workplace musculoskeletal problem, accounting for almost half of those reporting a problem in 12 months (47.9%; *n* = 34/71). Overall, 294 problems were reported in 12 months and 99 in 7 days, with 84.5% (*n* = 60/71) of students experiencing problems at multiple sites within 12 months and 76.5% (*n* = 26/34) within 7 days. The mean number of concurrent areas of problems reported by students in the 12 months and 7 day recall periods were 4.4 (±2.9) and 3.1 (±1.6) respectively.

**Fig. 1:**
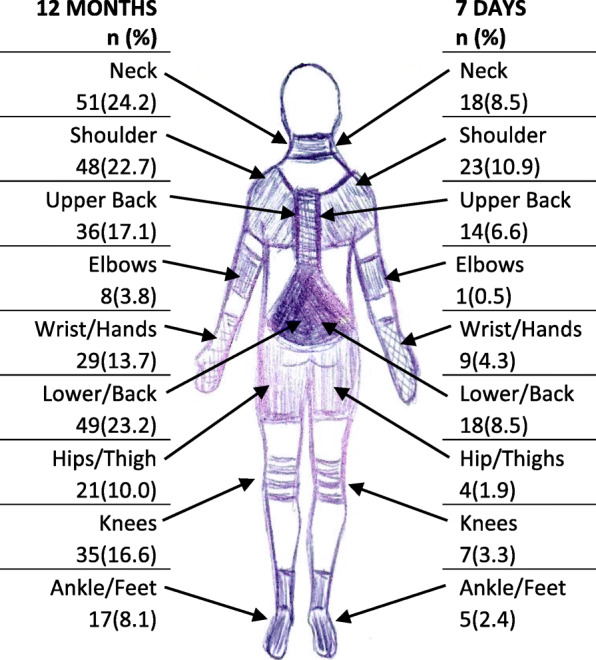
Prevalence of reported musculoskeletal problems by body region in the 12 month and 7 day recall period in the total survey sample (*n* = 211)

In the total survey sample (*n* = 211), the more prevalent reported sites of workplace musculoskeletal problems in the 12 month recall period (Fig. 1) were the neck (24.2%), lower back (23.2%), shoulders (22.7%) and upper back (17.1%). The shoulders (10.9%), lower back (8.5%), neck (8.5%), and upper back (6.6%) were also the most common body areas reported in 7 days (Fig. 1).

### Reported workplace musculoskeletal problems that prevented activity and needed medical assistance

When workplace musculoskeletal problems were reported, the most common problems preventing activities within the 12 months recall period were the lower back (23.9%; *n* = 17/71), knees (21.1%; *n* = 15/71), shoulders and upper back (15.5%; *n* = 11/71). In the 7 day recall period, the most common workplace musculoskeletal problems preventing activities occurred in the shoulders (20.6%; *n* = 7/34), wrists/hands, upper and lower back (14.7%; *n* = 5/34) and neck (11.8%; *n* = 4/34; Table 2).

**Table 2:** Prevalence of reported musculoskeletal problems, prevention of activity and needs to seek attention in the sample reporting a problem in the 12 months (*n* = 71) and 7 day (*n* = 34) recall period

Body area	Within last 12 months			Within last 7 days		
	Total Problems n (%)	Problems preventing daily activities n (%)	Problems requiring physician or health professional assistance n (%)	Total Problems n (%)	Problems preventing daily activities n (%)	Problems requiring physician or health professional assistance n (%)
Neck	51(71.8)	9(12.7)	21(29.6)	18(52.9)	4(11.8)	5(14.7)
Shoulders	48(67.6)	11(15.5)	23(32.4)	23(67.6)	7(20.6)	6(17.6)
Upper back	36(50.7)	11(15.5)	21(29.6)	14(41.2)	5(14.7)	3(8.8)
Elbows	8(11.3)	4(5.6)	8(11.3)	1(2.9)	1(2.9)	1(2.9)
Wrists/hands	29(40.8)	7(9.9)	11(15.5)	9(26.8)	5(14.7)	2(5.9)
Lower back	49(69.0)	17(23.9)	21(29.6)	18(52.9)	5(14.7)	3(8.8)
Hips/thighs	21(29.6)	7(9.9)	9(12.7)	4(11.8)	2(5.9)	1(2.9)
Knees	35(49.3)	15(21.1)	23(32.4)	7(20.6)	1(2.9)	0(0.0)
Feet/ankles	17(23.9)	8(11.3)	9(12.7)	5(14.7)	1(2.9)	0(0.0)

The most common body areas requiring assistance from a physician or health professional in 12 months were the shoulders and knees (32.4%; *n* = 23/71), followed by the neck, upper and lower back (29.6%; *n* = 21/71) and wrists/hands (15.5%; *n* = 11/71). The shoulders (17.6%; *n* = 6/34) were the most common body area reported to require assistance from a physician or health professional in 7 days, followed by the neck (14.7%; *n* = 5/34), upper and lower back (8.8%; *n* = 3/34) and wrists/hands (5.9%; *n* = 2/34; Table 2).

### Factors associated with workplace musculoskeletal problems in occupational therapy students

In unadjusted models, female gender (odds ratio (OR) = 2.45, *p* = 0.046, 95% confidence interval (CI) = 1.02–5.91), previous musculoskeletal problems (OR = 4.60, *p* < 0.001, 95% CI = 2.50–8.44) and non-standard joint mobility (OR = 2.26, *p* = 0.010, 95% CI = 1.22–4.17) were associated with workplace musculoskeletal problems over 12 months (Table 3).

**Table 3:** Factors Influencing Self-Reported Workplace Musculoskeletal Problems in the 12 month Recall Period

Explanatory Variables	Workplace Musculoskeletal Problems In 12 months		Unadjusted Results			Adjusted Results		
Categorical Variables	Yes n (%)	No n (%)	***P***-value	OR	(95% CI)	***P***-value	OR	(95% CI)
Gender			0.046			0.018		
Male ^a^	7 (19)	29 (81)		1.000			1.000	
Female	65 (37)	110 (63)		2.448	[1.015, 5.905]		3.115	[1.219, 7.960]
Previous Musculoskeletal Problem/s			< 0.001			< 0.001		
No ^a^	27 (21)	102 (79)		1.000			1.000	
Yes	45 (55)	37 (45)		4.595	[2.503, 8.435]		5.030	[2.691, 9.405]
Joint Mobility			0.010			–		
Within Normal Limits^a^	43 (29)	107 (71)		1.000	[1.220, 4.170]		–	–
Abnormal Limits	29 (48)	31 (51)		2.255			–	
Overall Working Posture			0.961			–		
Good ^a^	46 (34)	88 (66)		1.000	[0.560, 1.839]		–	–
Poor	26 (35)	49 (65)		1.015			–	–
Stage of Study			0.537			–		
Early ^a^	32(32)	68 (68)		1.000			–	–
Late	40 (36)	71 (64)		1.197	[0.676, 2.120]		–	–
Participates in Exercises During Work-Based Training			0.277			–		
No ^a^	46 (32)	99 (82)		1.000			–	–
Yes	26 (39)	40 (61)		1.399	[0.764, 2.562]		–	–
Patient Caseload			0.510			–		
Physical ^a^	62 (33)	124 (67)		1.000			–	–
Pschosocial	20 (40)	15(60)		1.333	[0.566, 3.139]		–	–
Works with Patients Outside of Study			0.255			–		
No ^a^	43 (31)	94 (69)		1.000			–	–
Yes	29 (39)	45 (61)		1.409	[0.781, 2.541]		–	–
**Continuous Variables**	**Mean (SD)**							
Number of Patients Treated During Work-Based Training	37.4 (64.3)		0.144	1.003	[0.999, 1.008]	–	–	–
Hours of Work-Based Training	261.9 (222.6)		0.133	1.001	[1.000, 1.002]	–	–	–
Age	23.6 (6.1)		0.818	0.994	[0.948, 1.043]	–	–	–
BMI	23.8 (4.1)		0.703	0.986	[0.918, 1.060]	–	–	–

^a^Reference Category

- Variables That Were Eliminated From The Model

Female gender and previous musculoskeletal problems were independently associated with reporting a workplace musculoskeletal problem over 12 months in the adjusted model. After adjusting for previous musculoskeletal problems, female students had 3.1 times greater odds, on average, of reporting a workplace musculoskeletal problem in 12 months than males (OR = 3.12, *p* = 0.018, 95% CI = 1.22–7.96). After adjusting for gender, participants who reported a previous musculoskeletal problem had 5.0 times greater odds, on average, of reporting a workplace musculoskeletal problem in 12 months than participants who did not report a previous musculoskeletal problem in the last 12 months (OR = 5.03, *p* < 0.001, 95% CI = 2.69–9.41).

In unadjusted models, previous musculoskeletal problems (OR = 3.58, *p* = 0.001, 95% CI = 1.66–7.72) and non-standard joint mobility (OR = 3.51, *p* = 0.001, 95% CI = 1.65–7.47) were associated with workplace musculoskeletal problems in 7 days (Table 4). Using backwards stepwise elimination, female gender, previous musculoskeletal problems, non-standard joint mobility and working in psychosocially focused caseloads were found to be independently associated with reporting workplace musculoskeletal problems in 7 days. The adjusted model included all variables independently associated with a workplace musculoskeletal problem in 7 days. After adjusting for these co-variates, female participants had 5.2 times greater odds, on average, of reporting a workplace musculoskeletal problem in the 7 day recall period than males (OR = 5.25, *p* = 0.034, 95% CI = 1.14–24.26). Participants who reported a previous musculoskeletal problem had 3.1 times greater odds, on average, of reporting a workplace musculoskeletal problem in 7 days than participants who did report a previous musculoskeletal problem (OR = 3.13, *p* = 0.006, 95% CI = 1.39–7.04). Participants with non-standard joint mobility had 3.8 times greater odds, on average, of reporting a workplace musculoskeletal problem in 7 days than participants who reported normal joint mobility (OR = 3.82, *p* = 0.002, 95% CI = 1.655–8.796). Participants working in psychosocially focused caseloads had 3.1 times greater odds, on average, of reporting a workplace musculoskeletal problem in 7 days than participants with a physical caseload (OR = 3.04, *p* = 0.044, 95% CI = 1.03–8.97).

**Table 4:** Factors Influencing Self-Reported Workplace Musculoskeletal Problems in the 7 day Recall Period

Explanatory Variables	Workplace Musculoskeletal Problems In 7 days		Unadjusted Results			Adjusted Results		
Categorical Variables	Yes n (%)	No n (%)	***P***-value	OR	(95% CI)	***P***-value	OR	(95% CI)
Gender			0.076			0.034		
Male ^a^	2 (6)	34 (94)		1.000			1.000	
Female	32 (18)	143 (82)		3.804	[0.869, 16.656]		5.249	[1.136, 24.258]
Previous Musculoskeletal Problem/s			0.001			0.006		
No ^a^	12 (9)	117 (91)		1.000			1.000	
Yes	22 (82)	60 (73)		3.575	[1.657, 7.715]		3.130	[1.391, 7.040]
Joint Mobility			0.001			0.002		
Within Normal Limits^a^	16 (11)	134 (89)		1.000			1.000	
Abnormal Limits	18 (30)	43 (70)		3.506	[1.646, 7.466]		3.815	[1.655, 8.796]
Overall Working Posture			0.214			–		
Poor ^a^	9 (12)	66 (88)		1.000			–	–
Good	25 (19)	109 (81)		1.682	[0.740, 3.822]		–	–
Stage of Study			0.676			–		
Early ^a^	15 (15)	85 (85)		1.000			–	–
Late	19 (17)	92 (83)		1.170	[0.559, 2.449]		–	–
Participates in Exercises during Work-Based Training			0.341			–		
No ^a^	21 (14)	124 (86)		1.000			–	–
Yes	13 (20)	53 (80)		1.448	[0.675, 3.106]		–	–
Patient Caseload			0.092			0.044		
Physical ^a^	27 (15)	159 (85)		1.000			1.000	
Psychosocial	7 (28)	18 (72)		2.290	[0.874, 6.003]		3.039	[1.030, 8.973]
Works with Patients Outside of Study			0.976			–		
No ^a^	22 (16)	115 (84)		1.000			–	–
Yes	12 (16)	62 (84)		1.012	[0.469, 2.181]		–	–
**Continuous Variables**	**Mean (SD)**							
Number of Patients Treated During Work-Based Training	37.4 (64.3)		0.755	0.999	[0.993, 1.005]	–	–	–
Hours of Work-Based Training	261.9 (222.6)		0.135	1.001	[1.000, 1.003]	–	–	–
Age	23.6 (6.1)		0.539	1.018	[0.962, 1.077]	–	–	–
BMI	23.8 (4.1)		0.930	0.996	[0.909, 1.092]	–	–	–

^a^Reference Category

- Variables That Were Eliminated From The Model

## Discussion

Workplace musculoskeletal disorders are a concern for individuals, the workforce, and the community due to inherent health, activity and economic impacts [1]. Health professionals are often at high risk of musculoskeletal problems, jeopardising the sustainability of the healthcare workforce [3]. This study revealed occupational therapy students were already at risk of musculoskeletal problems during their work-based undergraduate training. One in three occupational therapy students reported workplace musculoskeletal problems within a 12 month period. Almost half of the students reporting a musculoskeletal problem in 12 months, also reported a problem in 7 days.

While occupational therapy students only participate in work-based training for part of the year (up to a maximum of 10-week full-time blocks, with a maximum of 16 weeks, over two 8-week blocks in year 4), the prevalence of reported problems is around half of that reported by full-time practicing occupational therapists (63%) in the study by Passier and McPhail (2011) [15].

Early musculoskeletal problems may not necessarily be serious or on-going. However problems can be precursors to future, more serious disorders and often need to be addressed [1, 39]. Workplace musculoskeletal problems reported by occupational therapy students in this study were already impacting their daily activities, with many seeking medical attention. Students reported concurrent problems at multiple sites with 294 problems reported in 12 months and 99 problems in 7 days. Approximately one third of problems prevented daily activities. These findings are comparable with results from a study with practicing occupational therapists, indicating workplace musculoskeletal problems limit leisure (22.3%) and everyday activities (10.5%) [15].

Students sought treatment for around half of the reported problems in 12 months and a quarter of problems in 7 days. The proportion of practicing occupational therapists with workplace musculoskeletal problems who sought assistance/treatment (15%) was less than students in our study and may be in part due to the culture of self-treating in health professionals [15]. If students are more likely to seek treatment, this may be an opportunity for educational strategies to address workplace musculoskeletal risks and their management.

Differences in survey tools and definitions of workplace musculoskeletal disorders/problems between studies makes direct comparison difficult. Our study defined musculoskeletal problems as an ache, pain, numbness and discomfort, which are more prevalent than disorders or injuries to muscles, tendons, intervertebral discs and nerves [1, 39]. Considering this, the results of our study have primarily been compared with studies sharing similarity in data collection, design and reporting of workplace musculoskeletal problems in occupational therapists and students [13, 14, 25].

Similar to previous research with occupational therapists, the most common body areas for workplace musculoskeletal problems in occupational therapy students were the neck, lower back, shoulders, wrists/hands and the upper back [13–15]. The high prevalence of musculoskeletal problems in the upper extremities in occupational therapy students are consistent with previous studies and possibly related to caseload [13]. We found the prevalence of problems reported in the 7 day period to be more consistent with existing data in occupational therapists. This consistency in 7 day recall data with previous research may be related to a more accurate recall within a 7 day recall period rather than over 12 months. Students in first and fourth year, in particular, were participating in similar full-time hours to working therapists in the 7 day recall period.

Knee and ankle/foot problems were less commonly reported. The combined prevalence was 18.1% of total problems reported in 12 months and 12.1% in 7 days. However, lower limb problems in students were approximately double of those experienced by practicing occupational therapists in 12 months and overall throughout their career [15]. While some problems like knee problems were not as common as other areas, they were reported by almost half of students reporting a problem. In this study, among students with knee problems 32% sought treatment and 21% reported restrictions in their daily activities. Students indicated at least 34% of their work-based training involved standing or walking with around half of the students reporting participating in heavy physical caseloads (46.5%). Physically demanding caseloads [13] requiring prolonged weight-bearing may account for the nature, prevalence and severity of lower limb problems reported.

Occupational therapy students are exposed to the same workplace risks as occupational therapists while learning in real-world work settings with clients. In our study, 33.6% already report experiencing musculoskeletal problems. Students reporting problems is consistent with studies in nursing, dentistry, and medical science students reporting the prevalence of workplace musculoskeletal problems before graduation of 80% [28], 61% [29] and 38% [2] respectively. While there is higher prevalence in nursing and dentistry, this may, in part, be explained by ergonomically challenging and repetitive work practice in nursing and prolonged static positioning in dentistry. Medical science involves many desk-based tasks [2]. Desk or table top tasks in occupational therapy may contribute to the similarity in reported musculoskeletal problems (around a third of students) between disciplines.

Our data affirms that occupational therapy students are vulnerable to workplace musculoskeletal problems during their training. Smith et al. (2006) also found occupational therapy students to be at risk, reporting prevalence of upper body problems (75.5%) to be over double the prevalence of musculoskeletal problems in our students (33.6%). While Smith et al. (2006) had a higher prevalence of general upper body problems reported, our study was specific to reporting only workplace related musculoskeletal problems, which may explain this difference. The proportion of neck (71.8% vs 67.4%), shoulder (67.6% vs 46.3%) and upper back (50.7% vs 39.5%) problems between studies were more closely matched than overall prevalence [25]. However, the prevalence of musculoskeletal problems ranging from 33.6–77.5% in student therapists is a concern.

The proportion of occupational therapy students reporting workplace musculoskeletal problems in our study was at least half the proportion of early career practitioners reporting problems in occupational therapy (63%) [15] and podiatry (45%) [26]. Novice clinicians (≤5 years practice experience) have greater risk of workplace musculoskeletal problems [26], making this rate in students of greater concern. The lack of conditioning for full-time work and condensed time-limited placement blocks may exacerbate vulnerability to injury.

Risk factors associated with reported musculoskeletal problems were female gender, previous musculoskeletal problems, non-standard joint mobility and working in psychosocially focused caseloads. Female gender was associated with a 3 and 5 times greater odds of reporting a problem in the 12 month and 7 day recall periods respectively. This is consistent with other health workforce literature, and may be accounted for, in part, by inherent sex differences in physical capacity and size [19]. In a recent study of 95 practicing occupational therapists, 61% of females reported a workplace musculoskeletal disorder in comparison to 26% of males [13].

Having a previous musculoskeletal problem was associated with a 3 and 5 times greater odds of reporting a workplace problem in the 7 day and 12 month recall period respectively. Similar to our finding, previous studies have identified previous pain episodes as a risk factor for future musculoskeletal problems [40, 41]. Other personal risk factors previously identified in the literature may explain a person’s risk to both non-workplace and workplace musculoskeletal problems. These risk factors include co-morbidities, reduced rest, poor injury recovery, poor nutrition and fitness levels [18]. Another personal risk factor may be non-standard joint mobility which was found to be associated with reported problems (3 times greater odds in the 7 day recall).

Interestingly, working in psychosocially focused caseloads were associated with 3 times greater odds of reporting problems in the 7 day recall period. While traditional risk factors for workplace musculoskeletal disorders include ergonomic and physical demands, our results support emerging research about the psychological risk factors to musculoskeletal health [27]. Psychosocially focused caseloads can have greater psychological demands than physical caseloads [42]. Students working in psychosocially focused caseloads may experience a lack of social/emotional support, high job demands and reduced job control/autonomy [43]. Psychological demands place stress on the body, which may accumulate over time, leading to workplace musculoskeletal disorders [10, 44]. Psychosocial demands of monotonous work, low job control and high workload have been found to have long-term, cumulative effects on the developmental of musculoskeletal problems [45, 46]. Some occupational therapy students and practicing occupational therapists hold negative perceptions of psychosocial practice resulting in staff shortages that lead to increased pressure on staff [43]. This is a known risk factor for workplace musculoskeletal disorders [43]. Alternatively, higher likelihood of problems associated with psychosocial caseloads may be explained by occupational therapy students being more aware of biomechanical and ergonomic injury risk factors, with less focus on psychological risk factors, such as their own mental health, and personal resilience.

Data in Australia shows a marked decline by approximately 20% in occupational therapy registrations starting between ages 25–29 years, and continuing to decline thereafter [47]. While many reasons for this decline might exist, workplace musculoskeletal problems may limit the career-span of therapists, and the profession risks losing its experienced workforce. The healthy worker effect, whereby workers leave the profession early in their career due to injury or illness, leaving a cohort of healthy older workers [48], may explain some variance in prevalence of musculoskeletal problems between practicing therapists (23–63%) [9–16] and students (33.6%) in this study. Ergonomic education and resilience training as a part of the university curriculum, alongside practical on-site training, are possible strategies to mitigate musculoskeletal risks and impact on students.

### Future work

Investigating educational and practical interventions to reduce ergonomic risk factors and increase mental health resilience in occupational therapy students is important. Possible interventions for evaluation may include ergonomic assessment and intervention for tasks commonly completed by occupational therapists, musculoskeletal health education in university curricula and workplaces, and on-going mental health resilience training.

### Limitations

Several limitations are noted. Direct contact in practicum classes was only made with first and fourth-year students with class lists used to email all students in second and third-year. While the response rate is reasonable (53%) and higher than previous studies (33%) [11, 15] this may affect the accuracy and validity of the results. However, given the sample size calculation of 202, including 211 participants fits within the requirements to estimate the prevalence of musculoskeletal problems in approximately one third of the population (± 6.5%). It is possible that students with musculoskeletal problems are more likely to respond to the survey [15] and/or reach the screener question, introducting a potiential bias. However, 66% of students who completed the survey did not report any workplace musculoskeletal problems.

The findings are consistent with other studies on prevalence of musculoskeletal problems in health professionals, but a background rate of problems in the sample, consistent with the general population, is likely. This was addressed by collecting data specific to work-related musculoskeletal problems and previous non work-related musculoskeletal problems. As non work-related musculoskeletal problems increased the odds of reporting work-related musculoskeletal problems, it is possible some people in the population were more susceptible to these issues.

Previous literature has indicated that the prevalence of workplace musculoskeletal problems may differ between contexts [14]. This study was limited by only surveying students from one learning context, whereby the results may not be generalisable to other universities or geographical locations.

The recall periods of 12 months and 7 days are possible limitations as some students were not contacted immediately post work-based training due to the availability of students and the timing of work-based training. Previous studies demonstrate that the 12 month recall period is likely to result in recall bias [49] and may also have been negatively impacted upon by occupational therapy students not participating in work-based training year-round, however, the results would likely be conservative.

All data including musculoskeletal problems and arthrometric data were self-reported which may have resulted in reporting bias or inaccuracies [14]. Students may have reported problems that were not related to work-based training or problems that did not match the definition. Qualitative studies to understand the impact of workplace musculoskeletal problems on occupational therapy students is warranted. Finally, causation of musculoskeletal problems could not be determined due to the cross-sectional study design [50].

## Conclusions

Occupational therapy students, prior to graduation, already experienced a high prevalence of workplace musculoskeletal problems during their work-based training in this study. Workplace musculoskeletal problems reported by student therapists is of concern as these problems are often precursors to more significant workplace musculoskeletal disorders and jeopardise the sustainability of the occupational therapy workforce.

High prevalence of musculoskeletal problems in this study calls training programs and researchers to action. Further work is needed to identify strategies to protect the musculoskeletal health of occupational therapy students and new graduates. Training programs should consider providing more ergonomic, manual handling and mental health resilience training to occupational therapy students prior to attending work-based training. Educators need to challenge misconceptions that students in physical caseloads are at higher risk of workplace musculoskeletal problems compared to students working in psychosocially focused caseloads.

## Data Availability

The datasets used and/or analysed during the current study are not available publicly as this was not specified in the original ethical approval request, however, may be available in part from the corresponding author upon reasonable request.

## Abbreviations

SNMQ: Standardised Nordic Musculoskeletal Questionnaire
SD: Standard Deviation
IQR: Interquartile Range
*n*: Number
OR: Odds Ratio
CI: Confidence Interval
